# 8-Oxoguanine accumulation in mitochondrial DNA causes mitochondrial dysfunction and impairs neuritogenesis in cultured adult mouse cortical neurons under oxidative conditions

**DOI:** 10.1038/srep22086

**Published:** 2016-02-25

**Authors:** Julio Leon, Kunihiko Sakumi, Erika Castillo, Zijing Sheng, Sugako Oka, Yusaku Nakabeppu

**Affiliations:** 1 Division of Neurofunctional Genomics, Department of Immunobiology and Neuroscience, Medical Institute of Bioregulation, Kyushu University, 3-1-1 Maidashi, Higashi-ku, Fukuoka 812-8582, Japan

## Abstract

Oxidative stress and mitochondrial dysfunction are implicated in aging-related neurodegenerative disorders. 8-Oxoguanine (8-oxoG), a common oxidised base lesion, is often highly accumulated in brains from patients with neurodegenerative disorders. MTH1 hydrolyses 8-oxo-2′-deoxyguanosine triphosphate (8-oxo-dGTP) to 8-oxo-dGMP and pyrophosphate in nucleotide pools, while OGG1 excises 8-oxoG paired with cytosine in DNA, thereby minimising the accumulation of 8-oxoG in DNA. *Mth1/Ogg1*-double knockout (TO-DKO) mice are highly susceptible to neurodegeneration under oxidative conditions and show increased accumulation of 8-oxoG in mitochondrial DNA (mtDNA) in neurons, suggesting that 8-oxoG accumulation in mtDNA causes mitochondrial dysfunction. Here, we evaluated the contribution of MTH1 and OGG1 to the prevention of mitochondrial dysfunction during neuritogenesis *in vitro*. We isolated cortical neurons from adult wild-type and TO-DKO mice and maintained them with or without antioxidants for 2 to 5 days and then examined neuritogenesis. In the presence of antioxidants, both TO-DKO and wild-type neurons exhibited efficient neurite extension and arborisation. However, in the absence of antioxidants, the accumulation of 8-oxoG in mtDNA of TO-DKO neurons was increased resulting in mitochondrial dysfunction. Cells also exhibited poor neurite outgrowth with decreased complexity of neuritic arborisation, indicating that MTH1 and OGG1 are essential for neuritogenesis under oxidative conditions.

Mitochondrial dysfunction associated with oxidative stress has been implicated in ageing-related neurodegenerative disorders such as Alzheimer’s (AD)^1^,^2^, Parkinson’s (PD)^3^ and Huntington’s diseases (HD)^4^. Reactive oxygen species (ROS) are inevitably produced as by-products of the mitochondrial electron transport chain-oxidative phosphorylation system that drives ATP synthesis, and ROS production is markedly accelerated by mitochondrial dysfunction^5^,^6^.

ROS are highly reactive molecules that can attack various biomolecules such as lipids, proteins, and nucleic acids. Among all nucleobases, guanine is the most susceptible to oxidation by ROS^7^ resulting in the generation of 8-oxoguanine (8-oxoG). 8-oxoG is the major oxidised base in both nucleotide pools and polymerised DNA or RNA^8^,^9^. We and others have shown that 8-oxoG is highly accumulated in AD, PD and HD^10^,^11^,^12^,^13^. 8-oxoG can pair with adenine as well as cytosine, thereby causing G to T transversion mutations^14^. The level of 8-oxoG in DNA is kept low mainly by two enzymes: MTH1 (also known as NUDT1) and OGG1^9^,^14^. MTH1 hydrolyses 8-oxo-2′-deoxyguanosine triphosphate (8-oxo-dGTP) to 8-oxo-dGMP and pyrophosphate, thus preventing incorporation of 8-oxo-dGMP into nascent DNA by DNA-polymerases. OGG1 excises 8-oxoG opposite cytosine in DNA to initiate a base excision repair process. MTH1 and OGG1 both localize in nuclei and mitochondria as well as in cytoplasm^15^,^16^,^17^,^18^.

MTH1 or OGG1-defective mice accumulate 8-oxoG in mitochondrial DNA (mtDNA), thus resulting in mitochondrial dysfunction and cell death^19^,^20^,^21^,^22^. Using *Mth1*/*Ogg1*-double knockout (TO-DKO) mice, we previously demonstrated that accumulation of 8-oxoG in mtDNA and nuclear DNA of neurons and microglia causes neurodegeneration with prominent neuronal loss and microgliosis^16^. For neurons, maintenance of mtDNA integrity is essential for the continuous supply of energy to maintain membrane potentials, neuronal signalling and synaptic plasticity^23^, as well as to prevent neuronal death. In addition to maintaining neuronal functions in mature neurons, mitochondrial function is essential for the differentiation and maturation of neurons during development, including the high energy-demanding events of neuritogenesis and branching of axons or dendrites. Indeed, it has been shown that ATP supply from mitochondria is essential for neuritogenesis *in vitro*^24^, and also that mitochondrial activation plays a key role in pituitary adenylate cyclase-activating polypeptide-mediated neurite outgrowth^25^.

The mitochondrial nucleotide pool and mtDNA are always exposed to ROS, suggesting that defence mechanisms minimising the accumulation of oxidative lesions in mtDNA are important during neuritogenesis. Because mtDNA is particularly susceptible to the accumulation of 8-oxoG, we hypothesised that MTH1 and OGG1 function cooperatively to keep mtDNA levels of 8-oxoG low, thereby preventing mitochondrial dysfunction to ensure proper neuritogenesis. To test our hypothesis, we isolated cortical neurons from adult wild-type and TO-DKO mouse brains and cultured them *in vitro*. We examined the extent of neuritogenesis in the presence or absence of antioxidants in B27 supplements. TO-DKO neurons exhibited remarkably poor neuritogenesis in the absence of antioxidants with increased accumulation of 8-oxoG in mtDNA and significantly reduced mitochondrial membrane potential (MMP), especially in neuritic mitochondria, in comparison to wild-type neurons. We thus demonstrated that MTH1 and OGG1 play essential roles in the defence of mtDNA during neuritogenesis.

## Results

### MTH1 and OGG1 proteins in adult mouse brain

**Figure 1:**
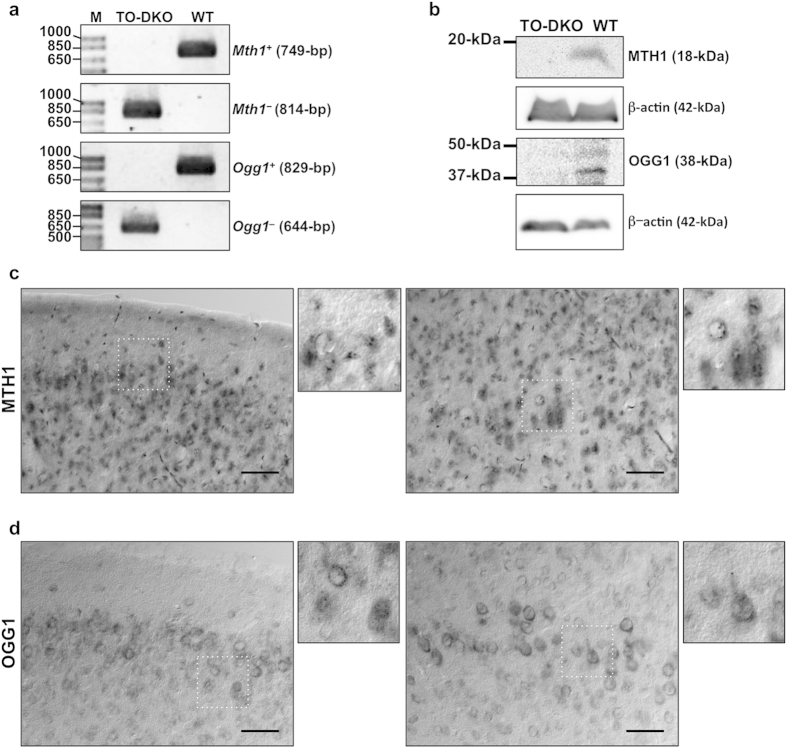
Localisation of MTH1 and OGG1 proteins in adult mouse cerebral cortex. (**a**) Genotyping of wild-type and *Mth1*/*Ogg1*-double knockout (TO-DKO) mice. Wild-type (*Mth1*^+^, *Ogg1*^+^) and mutant (*Mth1*^−^, *Ogg1*^−^) alleles were amplified using specific primer sets. (**b**) Cortical tissues from wild-type and TO-DKO male mice were subjected to western blotting with antibodies against MTH1, OGG1, and β-actin as a loading control. (**c**) Immunohistochemical detection of MTH1 in wild-type neocortex, layers 1–3 (left panel) and layers 4–5 (right panel). (**d**) Immunohistochemical detection of OGG1 in wild-type neocortex, layers 1–3 (left panel) and layers 4–5 (right panel). Scale bar = 50 μm.

TO-DKO and wild-type mice were generated by mating *Mth1*^+*/*−^*Ogg1*^+*/*−^ double-heterozygous mice and were genotyped by PCR (Fig. 1a). Extracts prepared from cortices of adult wild-type and TO-DKO brains were subjected to western blotting using anti-MTH1 and anti-OGG1 antibodies (Fig. 1b). An 18 kDa protein corresponding to MTH1, and a 38 kDa protein corresponding to OGG1 were detected only in wild-type extracts, confirming that the cortex of the TO-DKO mouse is completely deficient in both MTH1 and OGG1 proteins.

To visualise MTH1 or OGG1 proteins in brain regions, we performed immunohistochemistry using coronal sections of adult brains prepared from wild-type and TO-DKO mice. High levels of both MTH1 and OGG1 were detected in neocortex, hippocampal formation and hypothalamus and in subregions of the thalamus (Supplementary Fig. S1). In the neocortex, MTH1 was homogeneously localised in layers 2 to 3 and 4 to 6 most neurons and glia, exhibiting a punctate nuclear and a diffuse cytoplasmic or neuropil immunoreactivity (Fig. 1c). OGG1 was heterogeneously localised in layers 2 to 3 and 4 to 6, mainly in the retrosplenial, piriform and amygdala areas (Supplementary Fig. S1b) and exhibited a perinuclear and diffuse cytoplasmic or neuropil immunoreactivity (Fig. 1d). These results indicate that MTH1 and OGG1 are expressed in most neurons in the mouse neocortex, with some differences in their level of expression.

### Cortical neurons isolated from adult wild-type and *Mth1/Ogg1*-DKO brains regenerate neuritis

**Figure 2:**
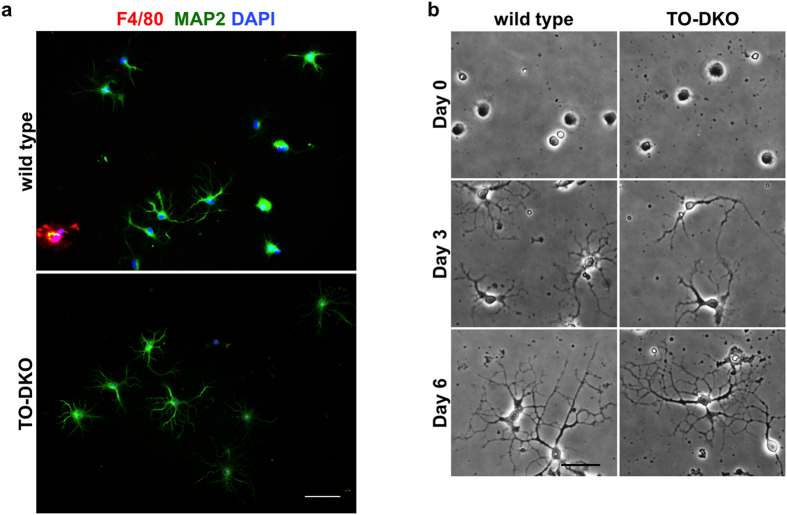
Cortical neurons isolated from adult wild-type and *Mth1/Ogg1*-DKO brains regenerate neurites. (**a**) Purity of cortical neuron culture at day 3 in vitro. Neuronal (MAP2, green) and microglial (F4/80, red) markers were detected by immunofluorescence microscopy. Nuclei were counter stained by DAPI (blue). About 95% of the cultured cells were MAP2-positive neurons. Merged images are shown. Scale bar = 50 μm. (**b**) Phase contrast images of cortical neurons isolated from adult wild-type (left panels) and *Mth1/Ogg1*-DKO (TO-DKO) (right panels) mice cultured for 0, 3 and 6 days, showing time-dependent neurite regeneration. Scale bar = 20 μm.

We isolated cortical neurons from wild-type and TO-DKO adult brains (15–19 weeks of age) to a purity higher than 95% (Fig. 2a) using density gradient separation as described in the Methods. These neurons were plated on coated glass substrates in Neurobasal medium supplemented with B27 containing antioxidants, and cultured for up to 6 days (Fig. 2b). Neurites grew from TO-DKO and wild-type cortical neurons within 3 days and highly extended neurites with extensive arborisation were present at day 6. We found no apparent morphological difference between wild-type and TO-DKO neurons (Fig. 2b, Day 6).

### Neurite extension and arborisation in *Mth1/Ogg1*-DKO cortical neurons were significantly impaired in the absence of antioxidants

**Figure 3:**
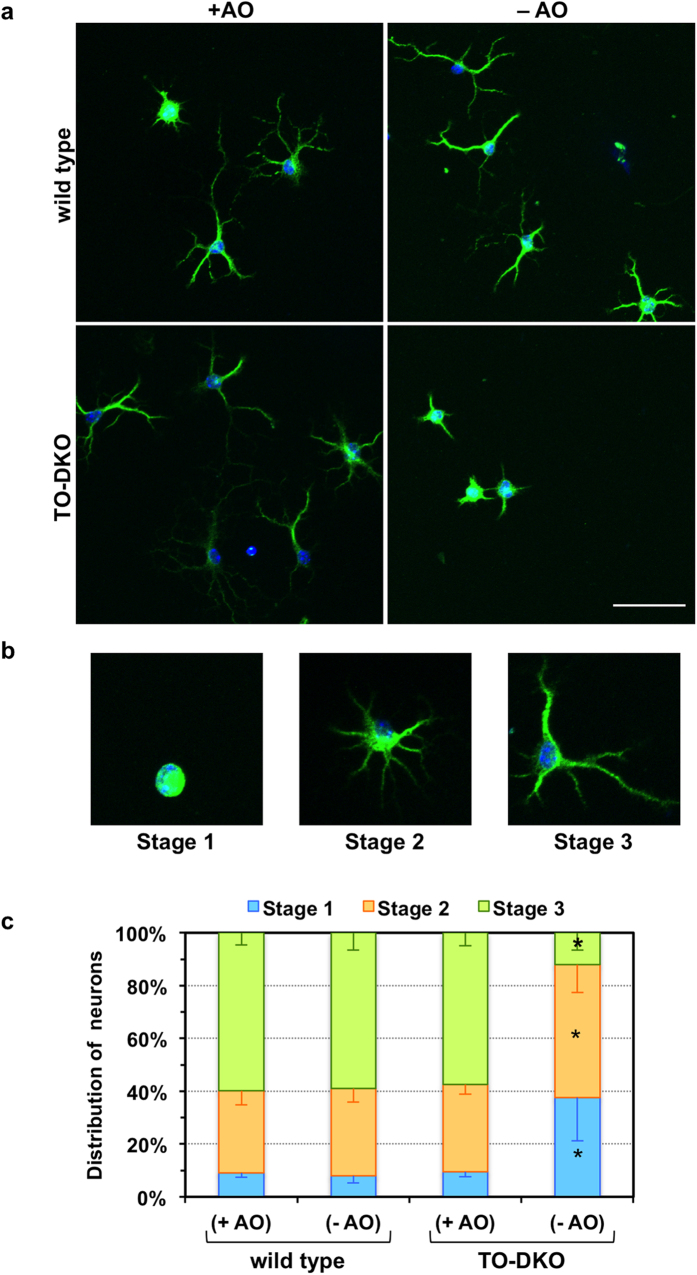
Neuritogenesis in cortical neurons isolated from adult *Mth1/Ogg1*-DKO but not wild-type mice was significantly impaired in the absence of antioxidants. (**a**) Adult cortical neurons isolated from *Mth1/Ogg1*-DKO (TO-DKO) and wild-type mice were cultured for 2 days in the absence (−AO) or presence (+AO) of antioxidants in B27 supplements, and were subjected to MAP2-immunofluorescence microscopy. Green: MAP2, blue: DAPI. Representative merged images are shown. Scale bar = 50 μm. (**b**) Regenerating neurons were classified into three stages: stage 1, lacking neurites; stage 2, with one or more minor neurite; stage 3, with one neurite at least twice as long as any other^26^. Representative images of neurons in each stage are shown. (**c**) Distribution of regenerating neurons. Percentage of neurons in each stage is shown. Error bar = SEM. N = 4 independent experiments. More than 50 neurons in each culture condition were examined. *Fisher’s exact test: p < 0.0001 vs. other sample in each stage.

To determine the vulnerability of TO-DKO cortical neurons to oxidative stress during neuritogenesis, we cultured these and wild-type neurons in medium supplemented with B27 lacking antioxidants (−AO) and compared neuritogenesis between the two groups. At the second day *in vitro* (2 DIV), neurons were visualised by microtubule-associated protein 2 (MAP2)-immunofluorescence microscopy (Fig. 3a). Based on the pattern of neurite outgrowth, regenerating neurons were classified into three stages: stage 1, lacking neurites; stage 2, with one or more minor neurite; stage 3, with one neurite at least twice as long as any other (Fig. 3b)^26^. For wild-type neurons cultured in either presence or absence of antioxidants and TO-DKO neurons cultured only in the presence of antioxidants, 57–60% were in stage 3, 31–33% were in stage 2, and less than 10% were in stage 1. In the absence of antioxidants, however, numbers of TO-DKO neurons in stage 3 were significantly decreased to 12%, while populations of neurons in stage 2 (50.4%) and stage 1 (37.6%) were significantly increased (Fig. 3c).

**Figure 4:**
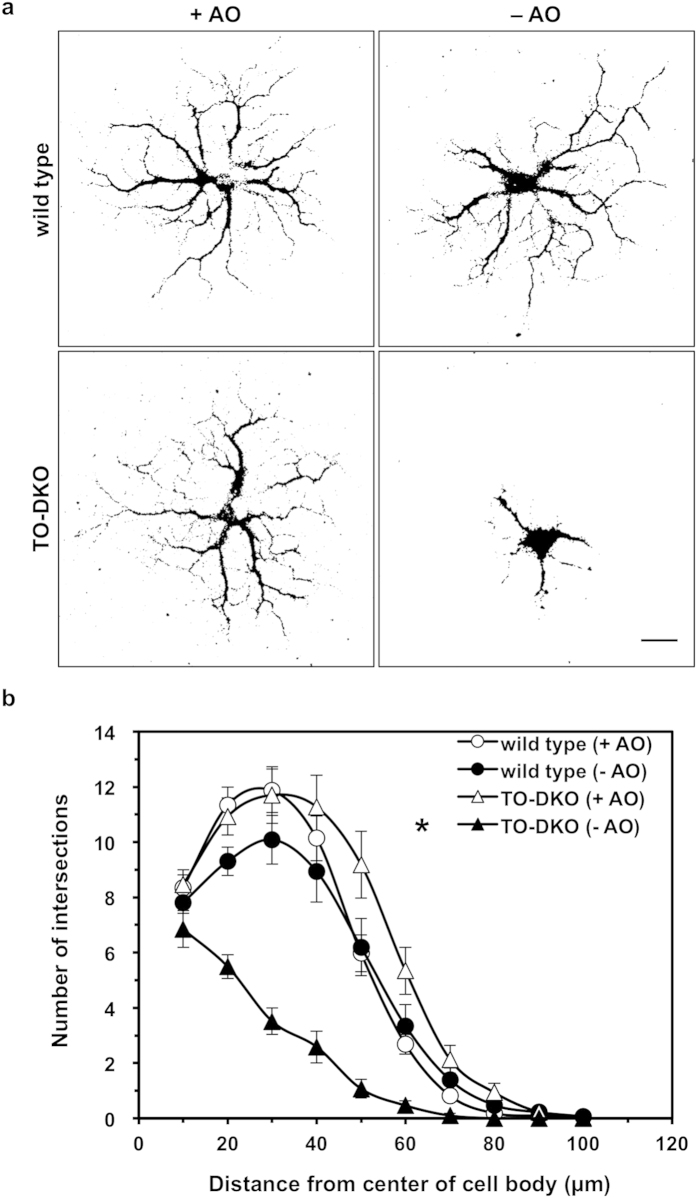
Neurite arborisation of cortical neurons isolated from adult *Mth1/Ogg1*-DKO but not wild-type mice was significantly impaired in the absence of antioxidants. (**a**) Adult cortical neurons isolated from *Mth1/Ogg1*-DKO (TO-DKO) and wild-type mice were cultured for 5 days in the absence (−AO) or presence (+AO) of antioxidants and were subjected to MAP2-immunofluorescence microscopy. Representative images are shown, indicating a simpler neuritic branching pattern in neurons from TO-DKO mice cultured in the absence of antioxidants. Scale bar = 20 μm. (**b**) Sholl’s concentric sphere analysis shows severely impaired neurite arborisation in TO-DKO neurons in the absence of antioxidants. More than 16 neurons were examined in each culture condition. Combined data from two independent experiments are shown. Error bar = SEM. MANOVA, between subjects: F(3, 171) = 19.671, p < 0.0001; within subjects: interaction, p < 0.0001 (Univar G-G, ε = 0.311). TO-DKO (−AO) vs. other three groups, *p < 0.0001.

At 5 DIV, most neurons from both wild-type and TO-DKO cortices extended multiple neurites up to 100 μm with many branches when cultured in the presence of antioxidants (Fig. 4a); however, the latter had fewer extensions and less complex neurite branching when cultured in the absence of antioxidants. We performed Sholl analysis to quantitatively compare the extension and arborisation of neurites (Fig. 4b). This also showed that TO-DKO neurons were significantly impaired in both neurite extension and arborisation in the absence of antioxidants.

**Figure 5:**
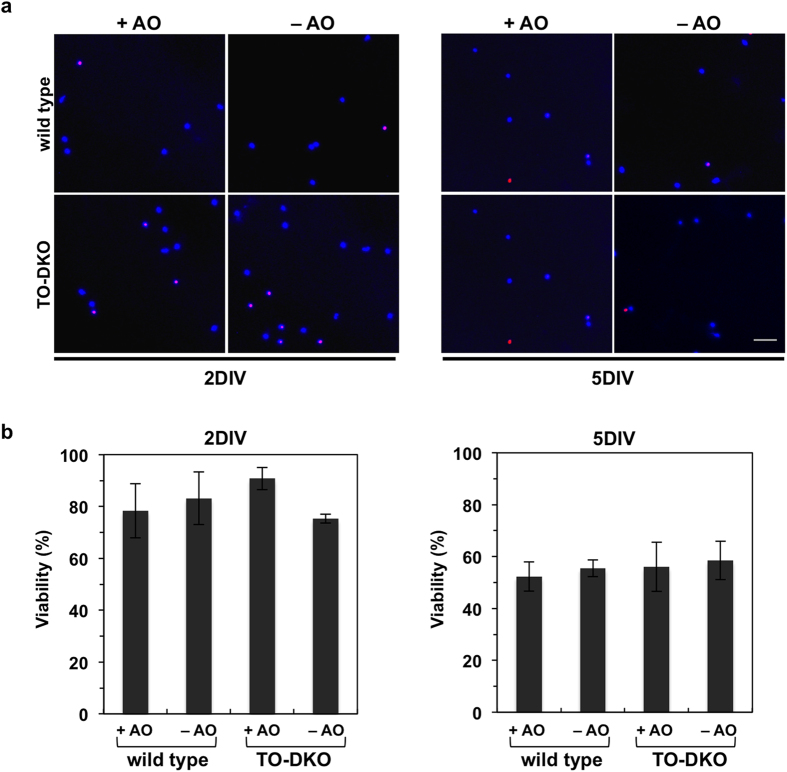
*Mth1/Ogg1*-DKO neurons do not exhibit increased cell death in the absence of antioxidants. (**a**) Adult cortical neurons isolated from adult *Mth1/Ogg1*-DKO (TO-DKO) and wild-type mice were cultured for 2 days *in vitro* (2DIV, left panels) and 5 days *in vitro* (5DIV, right panels) in the absence (−AO) or presence (+AO) of antioxidants in the B27 supplements, and were subjected to Hoechst 33258 (blue, nuclear staining) and propidium iodide (PI, red, dead cells) staining. Representative images are shown. (**b**) Viability. The percentage of PI-negative cells among Hoechst 33258-positive cells was determined. 2DIV (left, 2-way ANOVA, F(3, 16) = 0.7804, p = 0.522), and 5DIV (right, 2-way ANOVA, F(3, 16) = 0.1565, p = 0.924). Error bar = SEM. More than 11 cells in a given field were counted and five different fields per group (>79 cells/group) were examined. Scale bar = 40 μm.

Next, we examined cell viability to determine whether an absence of antioxidants in the B27 supplement induces cell death in TO-DKO neurons. Dead neurons were detected by propidium iodide (PI) staining (Fig. 5a). At 2DIV, in the presence of antioxidants, both wild-type and TO-DKO neurons exhibited 80 to 90% viabilities. In the absence of antioxidants, the viability of TO-DKO neurons often decreased to less than 80%; however, this decrease was not statistically significant among all samples. At 5DIV, the viabilities of both wild-type and TO-DKO neurons were decreased to approximately 50% regardless of the presence or absence of antioxidants in the media (Fig. 5b).

These results indicate that, in the absence of antioxidants, MTH1 and OGG1 are required for efficient *in vitro* neuritogenesis (extension and arborisation) but not for the viability of neurons.

### MTH1/OGG1 deficiency significantly increased the accumulation of 8-oxoguanine in mitochondrial DNA of cortical neurons cultured in the absence of antioxidants

**Figure 6:**
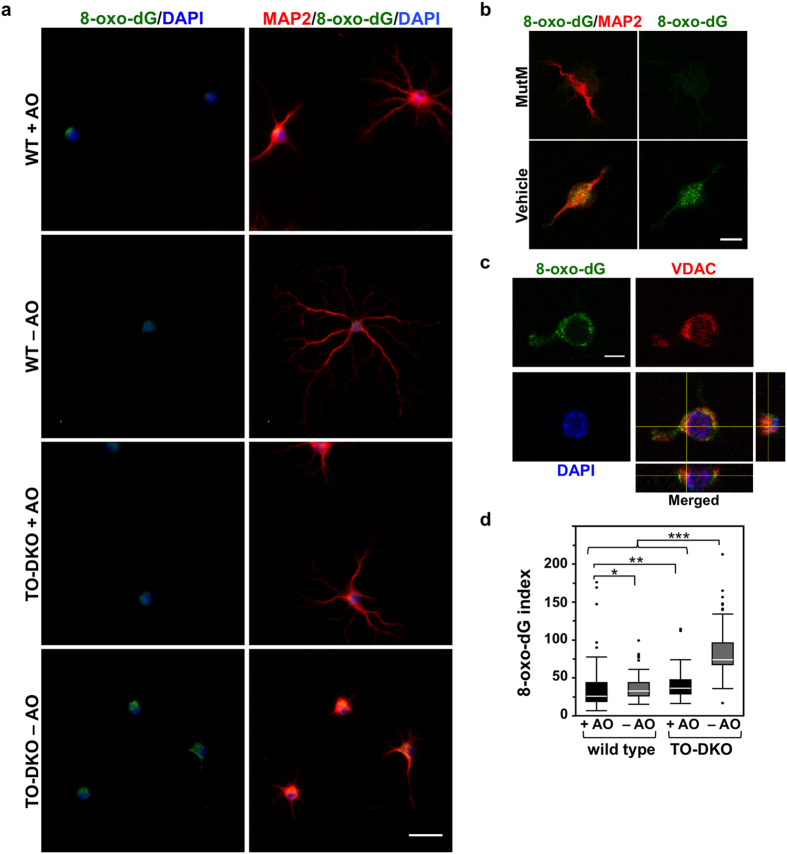
MTH1/OGG1 deficiency significantly increased accumulation of 8-oxoguanine in the mitochondrial DNA of cortical neurons in the absence of antioxidants. (**a**) 8-Oxo-deoxyguanosine (8-oxo-dG) detected in MAP2-positive neurons by immunofluorescence microscopy. Fixed neurons pre-treated with RNase were subjected to a mild denaturation with 25 mM NaOH before reacting with antibodies. Adult cortical neurons isolated from *Mth1/Ogg1*-DKO (TO-DKO) and wild-type (WT) mice were cultured for 2 days in the absence (−AO) or presence (+AO) of antioxidants. Green: 8-oxo-dG; red: MAP2: blue: DAPI. Scale bar = 20 μm. Cytoplasmic 8-oxo-dG immunoreactivity was increased in TO-DKO neurons maintained in the absence of antioxidant. (**b**) 8-Oxo-dG immunoreactivity in TO-DKO neurons in the absence of antioxidants was completely abolished by pre-treatment with MutM 8-oxoG DNA glycosylase. Scale bar = 10 μm. (**c**) Mitochondrial localization of 8-oxo-dG in a TO-DKO neuron. Immunofluorescence signals for mitochondrial voltage-dependent anion channel (VDAC, red) were co-localized with the cytoplasmic 8-oxo-dG immunofluorescence (green). Orthogonal views obtained by laser scanning confocal microscopy are shown. Blue: DAPI. Scale bar = 10 μm. (**d**) Quantitative evaluation of mitochondrial 8-oxo-dG in adult cortical neurons with (+AO) or without (−AO) antioxidants. More than 203 cells were examined for each group. 8-Oxo-dG indexes were calculated and are presented as whisker-box plots. Outliers are shown as dots. Wilcoxon/Kruskal–Wallis tests, chi square test p < 0.0001; post hoc comparison with Wilcoxon method, *p < 0.05, **p < 0.005, ***p < 0.0001.

To detect 8-oxoG accumulation in cellular DNAs of adult cortical neurons, we used an anti-8-oxo-dG antibody to perform immunofluorescence microscopy (Fig. 6). 8-oxoG in nuclear DNA can be detected only after pre-treatment with RNase and HCl^27^. We found no difference in nuclear 8-oxo-dG immunoreactivity between wild-type and TO-DKO neurons cultured with and without antioxidants (Supplementary Fig. S2). We then examined 8-oxo-dG immunoreactivity without HCl treatment, and found that cytoplasmic 8-oxo-dG immunoreactivity was significantly increased in TO-DKO neurons only when cultured in the absence of antioxidants (Fig. 6a). The 8-oxo-dG immunoreactivity detected in TO-DKO neurons cultured in the absence of antioxidants was abolished by pre-treatment with MutM 8-oxoG DNA glycosylase (Fig. 6b), and co-localised with the mitochondrial voltage-dependent anion channel (VDAC) (Fig. 6c), indicating that 8-oxoG was accumulated in mtDNA. The levels of 8-oxo-dG immunoreactivity in mtDNA were more than 2-fold higher in TO-DKO cortical neurons cultured in the absence of antioxidants, compared with other cells (Fig. 6d). Even in the presence of antioxidants, the 8-oxo-dG immunoreactivity was slightly but significantly higher in TO-DKO neurons than in wild-type neurons. 8-Oxo-dG immunoreactivity was detected in neurites as well as in cell bodies, especially in TO-DKO cortical neurons cultured in the absence of antioxidants.

### MTH1/OGG1 deficiency induced significant mitochondrial dysfunction in cortical neurons in the absence of antioxidants

**Figure 7:**
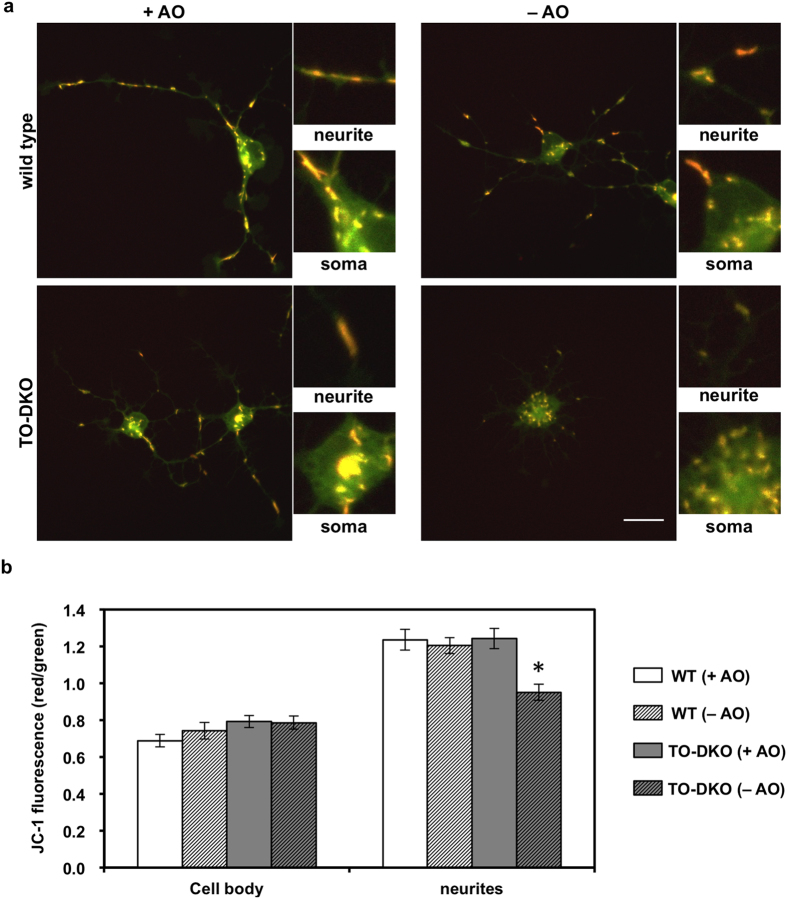
MTH1/OGG1 deficiencies cause mitochondrial dysfunction in adult cortical neurons. (**a**) Adult cortical neurons isolated from *Mth1/Ogg1*-DKO (TO-DKO) and wild-type brains were cultured for 2 days in the absence (−AO) or presence (+AO) of antioxidants and were then exposed to JC-1 dye. JC-1 dye emits green-fluorescence in its monomer form with lower membrane potential, and red-fluorescence in its aggregate form in normal mitochondria. Scale bar = 20 μm. (**b**) Red/green ratio of JC-1 fluorescence in cell body (soma in a) and neurites. Mitochondrial membrane potentials were significantly decreased in TO-DKO neurons cultured without antioxidants. 2-way ANOVA, F(3, 80) = 7.495, p = 0.0002 *Tukey-HSD test, TO-DKO (−AO) vs. other three groups. p < 0.002. More than 17 neurons were examined for each condition. Error bar = SEM.

To investigate whether accumulation of 8-oxoG in mtDNA of TO-DKO cortical neurons caused mitochondrial dysfunction, we first examined MMP in cortical neurons by confocal imaging of live cells labelled with the mitochondrial specific dye, JC-1, as a measure of the MMP. Under low MMP, JC-1 exists in its monomeric form emitting green fluorescence, whereas under high MMP, JC-1 forms aggregates which emit red fluorescence^28^. The ratio of red to green fluorescence was below 0.8 in cell bodies of both wild-type and TO-DKO neurons cultured with or without antioxidants (Fig. 7a,b). Interestingly, the ratio of red to green fluorescence in neurites was higher than 1.2 in both wild-type and TO-DKO neurons with antioxidants (Fig. 7a,b). However, in the absence of antioxidants, TO-DKO neurons exhibited a significantly lower ratio of red to green fluorescence, around 0.9. These results indicate that the MMP was significantly decreased in TO-DKO neurons without antioxidants, especially in mitochondria being transported in neurites.

**Figure 8:**
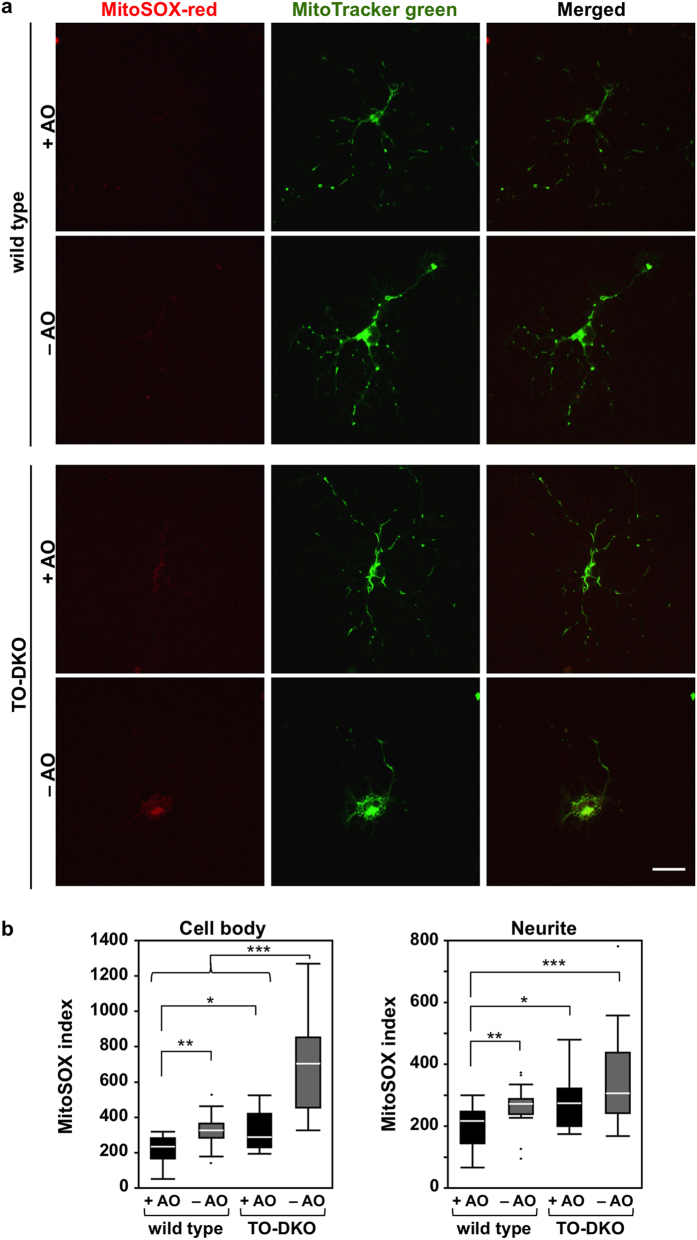
MTH1/OGG1-defciencies significantly increased mitochondrial production of superoxide in adult cortical neurons cultured in the absence of antioxidants. (**a**) Adult cortical neurons isolated from *Mth1/Ogg1*-DKO (TO-DKO) and wild-type brains were cultured for 2 days in the absence (−AO) or presence (+AO) of antioxidants, then incubated with MitoTracker (green) and MitoSOX (red). Representative images are shown. Scale bar = 20 μm. (**b**) MitoSOX index in cell body and neurites are shown as whisker-box plots. Mitochondrial superoxide production significantly increased in TO-DKO neurons cultured in the absence of antioxidants. More than 17 neurons were examined for each condition. Outliers are shown as dots. Wilcoxon/Kruskal–Wallis tests, chi square test p < 0.0001; post hoc comparison with Wilcoxon method, *p < 0.05, **p < 0.005, ***p < 0.0001.

Next, we examined mitochondrial superoxide production in each neuron using MitoSOX Red, a mitochondrial superoxide indicator, together with MitoTracker Green (Fig. 8). In cell bodies, wild-type neurons exhibited very low levels of MitoSOX Red fluorescence when cultured with or without antioxidants, while strong MitoTracker Green fluorescence was detected from cell bodies to extended neurites (Fig. 8a, top panels). TO-DKO neurons also exhibited low levels of red fluorescence and strong green fluorescence in cell bodies and extended neurites in the presence of antioxidants; however, TO-DKO neurons maintained in the absence of antioxidants, exhibited increased red fluorescence in cell bodies and in some neurites that was co-localised with strong green fluorescence (Fig. 8a, bottom panels). We quantified the MitoSOX Red intensity and normalised with respect to the mitochondrial area determined by MitoTraker Green. These quantitative data demonstrated that mitochondrial superoxide production in both cell bodies and neurites in wild-type neurons maintained without antioxidants was significantly higher compared with that in wild-type neurons maintained with antioxidants (Fig. 8b). In the presence of antioxidants, an increased mitochondrial superoxide production in both cell bodies and neurites was observed in TO-DKO neurons in comparison with wild-type neurons. When TO-DKO neurons were maintained in the absence of antioxidants, superoxide production was further increased by more than 2-fold in cell bodies and to a lesser extent in neurites, in comparison with TO-DKO neurons maintained with antioxidants (Fig. 8b).

### FCCP, an uncoupler of mitochondrial oxidative phosphorylation, inhibits neurite regeneration in wild-type cortical neurons in the presence of antioxidants

It is likely that TO-DKO neurons maintained in the absence of antioxidants accumulate significantly higher levels of 8-oxoG in mtDNA, leading to an impairment of the oxidative phosphorylation system and decreased ATP synthesis. To confirm that decreased ATP synthesis is sufficient to disturb neuritogenesis *in vitro*, we applied carbonyl cyanide-p-trifluoromethoxyphenylhydrazone (FCCP), which uncouples the electron transport chain from ATP synthesis, to wild-type neurons in the presence of antioxidants (Supplementary Fig. S3a). In the presence of 5 μM FCCP, neuritogenesis in wild-type neurons was significantly inhibited, indicating that uncoupling the oxidative phosphorylation system is sufficient to disturb neuritogenesis in adult cortical neurons (Supplementary Fig. S3b).

## Discussion

It has been well documented that MTH1 and OGG1 play essential roles in preventing 8-oxoG accumulation in nuclear DNA of proliferating cells and thus reduce mutagenesis and carcinogenesis^9^,^14^; however, their importance in neurons of the adult brain is poorly understood. In the present study, we showed that cortical neurons in adult mouse brain contain substantial levels of MTH1 and OGG1 proteins. We then demonstrated that MTH1 and OGG1 are essential for efficient neuritogenesis in adult cortical neurons *in vitro*, under oxidative conditions. To our knowledge, this is the first *in vitro* study of neuritogenesis in neurons isolated from adult mouse cortex.

MTH1/OGG1-deficient neurons maintained *in vitro* in the absence of antioxidants accumulate harmful levels of 8-oxoG exclusively in mtDNA and exhibit mitochondrial dysfunction and poor neuritogenesis. mtDNA encodes essential genes for the electron transport chain and oxidative phosphorylation system that drive ATP synthesis. Many reports have shown that damage to mtDNA causes mitochondrial dysfunction through altered mitochondrial gene expression resulting in functional impairments^29^ or mtDNA degradation^30^. Neurite outgrowth is known to be influenced by intracellular levels of calcium and ATP; both are tightly regulated by mitochondria. mtDNA depletion in embryonic hippocampal neurons by ethidium bromide results in suppression of axonogenesis with altered calcium homeostasis^31^. Also, a recent study using developing cerebellar Purkinje cells suggests that local ATP synthesis by dendritic mitochondria and ATP-phosphocreatine exchange are essential for dendritic development^24^. Local protein synthesis, actin filament and microtubule dynamics, membrane trafficking and phosphorylation reactions are key events for neuritogenesis that have high energy demands^26^,^32^,^33^,^34^,^35^,^36^. We suggest that mature cortical neurons in the adult brain may increase their energy demands to regenerate neurites when axons or dendrites are damaged *in vivo*; therefore, suppression of 8-oxoG accumulation in mtDNA by MTH1 and OGG1 may be crucial to maintain mitochondrial function to satisfy these demands.

ATP synthesis in mitochondria is always accompanied by ROS production, because electrons that leak from the electron transport chain reduce oxygen molecules to superoxide anions, which can be further converted into highly reactive ROS such as hydrogen peroxides or hydroxyl radicals. Up to 5% of oxygen consumed in mitochondria is converted to superoxide anions during normal mitochondrial respiration^5^. In the present study, we found that levels of mitochondrial superoxide were increased 1.5-fold in wild-type cortical neurons maintained in the absence of antioxidants compared with those maintained in the presence of antioxidants (Fig. 8), indicating that antioxidants in B27 supplement efficiently scavenge the ROS that are generated during normal mitochondrial respiration *in vitro*. Levels of mitochondrial superoxide in MTH1/OGG1-deficient neurons maintained in the presence of antioxidants were equivalent to those in wild-type neurons maintained in the absence of antioxidants, and were significantly increased by more than 2-fold in the absence of antioxidants (Fig. 8). In the presence of antioxidants, 8-oxoG levels in mtDNA were slightly but significantly higher in MTH1/OGG1-deficient neurons than in wild-type neurons (Fig. 6d). These results indicate that MTH1/OGG1 deficiency increases accumulation of 8-oxoG in mtDNA, thereby increasing mitochondrial ROS production even in the presence of antioxidants, probably because the slight increase in 8-oxoG levels in mtDNA is sufficient to disturb mitochondrial function. The mitochondrial ROS production in MTH1/OGG1-deficient neurons is further exacerbated in the absence of antioxidants, resulting in greater accumulation of 8-oxoG in mtDNA, thus escalating the vicious cycle.

The increased levels of mitochondrial ROS and 8-oxoG in mtDNA in MTH1/OGG1-deficient neurons maintained in the absence of antioxidants were mainly detected in cell bodies, with a slight increase in neurites (Figs 6 and 8). It is likely that neurite outgrowth from the cell body initiates in the proximity of mitochondria; therefore, the significant increase in ROS production in such mitochondria may also result in oxidation of other critical molecules such proteins and lipids to further exacerbate the impairment of neurite outgrowth in adult cortical neurons *in vitro*.

MMP was significantly decreased only in neuritic mitochondria in MTH1/OGG1-deficent neurons in the absence of antioxidants (Fig. 7), while MMP in cell body mitochondria was not altered. These results indicate that motile mitochondria in neurites are more susceptible to ROS-induced dysfunction than are mitochondria in the cell body (Fig. 8), even though the latter accumulate more 8-oxoG in mtDNA and produce more ROS. Biogenesis of mitochondria as well as their fusion and fission mainly take place in the cell body; therefore, mitochondria located in the cell body are capable of exchanging their mtDNA or other components among other mitochondria^6^, thus minimising mitochondrial dysfunction. However, mitochondrial fusion and fission may not occur among isolated mitochondria migrating across neurites, thus mitochondrial dysfunction persists in neuritic mitochondria.

It has been proposed that proper mitochondrial transport into neurites is tightly regulated to ensure the supply of ATP^23^,^37^; however, if these motile mitochondria are functionally impaired, they may not be able to meet the local energy demands. This may also be the cause of the severe impairment of neurite extension and arborisation observed in MTH1/OGG1-deficient neurons maintained in the absence of antioxidants. Uncoupling of oxidative phosphorylation and ATP synthesis by FCCP treatment reproduced impairment of neurite extension and arborisation in wild-type neurons in the presence of antioxidants (Supplementary Fig. S3), confirming that decreased ATP supply is responsible for of the impairment of neurite extension and arborisation observed in MTH1/OGG1-deficient neurons *in vitro* in the absence of antioxidants.

In the present study, we showed that impaired neurite extension and arborisation in MTH1/OGG1-deficient cortical neurons in the absence of antioxidants was accompanied by neuritic mitochondrial dysfunction, namely decreased MMP. These results suggest that neuritic mitochondria play an important role in neuritogenesis in adult cortical neurons; however, this is an apparent contradiction to observations in embryonic hippocampal pyramidal neurons^38^. Depletion of mitochondria in dendrites by overexpressing Mfn1, a GTPase mediating mitochondrial fusion, or TRAK2-MBD, a truncated form of motor adaptor protein that can disrupt mitochondrial transport, increased branching in the proximal portion of dendrites, indicating that dendritic mitochondria negatively regulate dendritic branching^38^. In the Mfn or TRAK2A-MBD overexpressing neurons, mitochondria are mainly localised in cell bodies without any dysfunction suggesting that these mitochondria may be able to provide sufficient energy to initiate branching in the proximity of dendrites. However, it is possible that mitochondria in embryonic hippocampal pyramidal neurons may have different roles in regulating dendritic branching in comparison to those in adult cortical neurons. Thus, further investigation is essential to delineate the difference.

We have previously shown that systemic administration of 3-nitropropionic acid to MTH1/OGG1-deficient mice causes early mitochondrial 8-oxoG accumulation in striatal neurons, resulting in severe mitochondrial dysfunction and calpain-dependent neuronal loss, which is further exacerbated by microgliosis^16^. In the present study, we observed no change in viability between wild-type and MTH1/OGG1-deficient neurons regardless of the presence or absence of antioxidants. In 3-nitropropic acid-administered MTH1/OGG1-deficient mice, the level of 8-oxo-dG in mtDNA in striatal neurons was increased by more than 40-fold compared with that in wild-type control mice^16^. However, in the present study, we observed an approximate 2-fold increase in the levels of 8-oxo-dG in mtDNA in MTH1/OGG1-deficient neurons in comparison to those in wild-type control (Fig. 6d) in the absence of antioxidants, indicating that mitochondrial dysfunction in MTH1/OGG1-deficient neurons maintained in the absence of antioxidants is very mild and does not induce cell death. In adult MTH1/OGG1-deficient mice, we have not observed any gross abnormality in their brains without any treatment^16^, however, we can not rule out the possibility of alteration in morphologies of their dendrites and axons during aging which is known to increase oxidative damage. Thus, morphological and functional alterations in aged MTH1/OGG1-deficient brains should be carefully examined. Moreover, it is well established that the microglial response *in vivo* also contributes to induction of neuronal death^16^, and thus *in vitro* co-cultured neurons and microglia exhibit significantly increased susceptibility to undergo neuronal death^39^. It would be interesting to examine whether MTH1/OGG1-deficient neurons co-cultured *in vitro* with microglia undergo neuronal death in the absence of antioxidants.

Our results shed new light on the essential functions of MTH1 and OGG1 during neuritogenesis in adult neurons *in vitro*. Future studies should investigate the *in vivo* roles of MTH1 and OGG1 in protecting mtDNA from oxidative damage, thereby maintaining brain functions under oxidative conditions, especially during the aging process.

## Materials and Methods

### Mice

We previously established *Mth1* and *Ogg1* gene knockout (KO) mice^40^,^41^. Heterozygous mice (*Mth1*^+*/*−^ and *Ogg1*^+*/*−^) have been backcrossed to C57BL/6J (Clea Japan, Tokyo, Japan) for more than 18 generations, thereby ensuring a standard C57BL/6J genetic background^14^. *Mth1*^+*/*−^*Ogg1*^+*/*−^ double-heterozygous mice obtained by crossing of *Mth1*^+*/*−^ and *Ogg1*^+*/*−^ mice were crossed to obtain *Mth1*^−*/*−^*Ogg1*^−/−^ (TO-DKO) and *Mth1*^+*/*+^*Ogg1*^+*/*+^ (wild-type) mice, and were inbred for two generations to obtain the mice used in each experiment. Wild-type and C57BL/6J mice were used as controls. For genotyping, specific primer sets were used. For the *Mth1*^+^ allele (749 bp): 5′-CTCTCCAGCCCTTGTTCAAGTTC-3′ and 5′-CCTACTCTCTTGGGCTTCATCC-3′; *Mth1*^−^ allele (814 bp): 5′-CTCTCCAGCCCTTGTTCAAGTTC-3′ and 5′-GAACCTGCGTGCAATCCATCTTGT-3′; *Ogg1*^+^ allele (829 bp): 5′-GTTAAGCTTCAAACGTGCCTC-3′ and 5′-GAAGGACTGTCCAGAAGCTA-3′; *Ogg1*^−^ allele (644 bp): 5′-GTTAAGCTTCAAACGTGCCTC-3′ and 5′-CTACGCATCGGTAATGAAGG-3′. All animals were maintained in a temperature-controlled (22 ± 2 °C, 55 ± 5% humidity), specific pathogen-free room with a 12 h light-dark cycle. The care and use of all animals were performed in accordance with prescribed national guidelines, and the Animal Care and Use Committee of Kyushu University granted ethical approval for the study.

### Primary culture of cortical neurons

We cultured cortical neurons isolated from 15–19-week-old wild-type and TO-DKO male mice following the protocol published by Brewer and Torricelli^42^ with slight modifications. Briefly, mice were euthanised by cervical dislocation, then brains were isolated and transferred to ice-cold Hibernate A (Life Technologies), supplemented with B27 and 0.5 mM glutamine (Life Technologies). The two hemi-cortices from each mouse were manually diced using a surgical blade (~0.5 mM thick) and then incubated in media containing 2 mg/ml papain in Hibernate A minus calcium at 32 °C on a linear shaker for 30 min. The digested tissue was transferred with a cut 1 ml tip to a medium free of papain and triturated 15 times using a siliconised Pasteur pipette. The triturated tissue was applied to the top of an OptiPrep 1.32 gradient (Sigma-Aldrich) and centrifuged at 800 × *g* for 15 min. Debris above 4 ml was discarded, and the fraction containing neurons was collected and diluted in 7 ml of Hibernate A/B27 with 0.5 mM glutamine. After centrifugation at 200 × *g* for 3 min, the pellet was resuspended in approximately 2 ml of conditional medium (Neurobasal A, 0.5 mM glutamine, 10 ng/ml fibroblast growth factor-2, 10 ng/ml brain-derived neurotrophic factor and penicillin/streptomycin), and viability was determined by the trypan blue exclusion test. For cell plating, 13 mm glass cover slips coated with 100 μg/ml poly-D-lysine and 5 μg/ml laminin were placed in 12 multi-well plates. Approximately, 1.5 × 10^4^ cells in 100 μl of conditioned medium were plated on each glass cover slip and allowed to attach for 1 h. Attached neurons were washed three times with supplement-free-Hibernate A at 37 °C and transferred to a 24-well plate containing 0.45 ml of conditioned medium equilibrated with 5% CO_2_ and supplemented with B27 containing antioxidants (superoxide dismutase, catalase, vitamin E, vitamin E acetate and glutathione) (B27 Supplement, Life Technologies), or lacking the antioxidants (B27 Supplement minus AO, Life Technologies), as described.

### Western Blotting

Cortical tissues were isolated from 10-week-old male mice and lysed in RIPA buffer (150 mM NaCl, 1% Nonidet P-40, 0.1% sodium deoxycholate, 0.1% SDS, 50 mM Tris-HCl pH 8.0), electrophoresed and blotted onto polyvinylidene difluoride or nitrocellulose membranes for detection of MTH1 or OGG1 respectively. The blots were blocked with 5% nonfat milk in Tris-buffered saline Tween 20 (TBST; 0.1% Tween 20 in 10 mM Tris-HCl, pH 7.5, 0.9% NaCl) for 1 h at room temperature. Rabbit anti-MTH1 antibody prepared previously^43^ was diluted to 0.5 μg/ml with 1% nonfat milk in TBST. Rabbit anti-OGG1 antibody (NB100-106, 1:5000, Novus Biologicals) was diluted with 3% nonfat milk in TBST. Primary antibodies were incubated for 12–16 h at 4 °C, followed by three 10 min washes with TBST. The blots were then incubated for 1 h at room temperature with horseradish peroxidase-linked protein A (Sigma-Aldrich) diluted in TBST (1:10000) for MTH1, and horseradish peroxidase-linked anti-rabbit IgG antibody (#7074, Cell Signalling, 1:2000) diluted with 3% nonfat milk in TBST for OGG1. The blot was washed three times for 10 min with TBST, incubated in Immobilon HRP substrate, and imaged on an Ez capture MG (ATTO, Tokyo, Japan).

### Immunofluorescence

To detect MAP2 or microglial marker, cells were fixed with 4% paraformaldehyde for 15 min at 37 °C, rinsed three times with phosphate-buffered saline (PBS) and stored at 4 °C until analysis. Cells were then permeabilised with 0.2% Triton X-100 in PBS for 10 min. After twice rinsing with PBS, cells were incubated in 10% goat serum/PBS at room temperature for 1 hr, followed by a single rinse with PBS. All primary antibodies were diluted in 1% goat serum/PBS and incubated with cells at 4 °C in a humidified chamber for 12–16 h. Corresponding secondary antibodies in 1% goat serum/PBS were then added and incubated for 1 h at room temperature. Cells were then washed once with 0.1% Tween 20/PBS for 10 min and twice with PBS for 5 min. Primary antibodies used were mouse anti-MAP2 (M4403, 1:1000, Sigma-Aldrich) and rat anti-F4/80 (CI:A3-1, 1:1000, SEROTEC). For detection of 8-oxoG in DNA, the protocols published by Ohno *et al.*^27^ were used with slight modifications. Cells fixed with 4% paraformaldehyde were permeabilised with 0.2% Triton X-100 in PBS for 10 min, then treated with RNase A at 37 °C for 1 h, followed by denaturation with ice-cold 25 mM NaOH in 50% ethanol to detect 8-oxoG in mtDNA, or by 2 N HCl to detect 8-oxoG in nuclear DNA. Cells were then incubated with mouse anti-8-oxo-dG (clone N45.1, 1:80, JaICA, NIKKEN SEIL, Fukuroi, Shizuoka, Japan), rabbit anti-VDAC (ab2877, 1:200, Abcam) or rabbit anti-MAP2 (AB5622, 1:1000, EMD Millipore). MutM protein treatment was applied to assess the specificity of the 8-oxo-dG signals prior to the denaturation step. Cells treated with RNase were incubated with 10 µg/ml of MutM protein (F3174, Fapy DNA glycosylase, Sigma-Aldrich) in nicking buffer [10 mM Tris-HCl (pH 7.5), 5 mM ZnCl_2_, 0.5 mM DTT, 0.5 mM EDTA, 1.5% glycerol, 100 µg/ml BSA] for 1 h at 37 °C. Secondary antibodies, goat anti-mouse-IgG Alexa 488, goat anti-rabbit-IgG Alexa 594, and goat anti-rat-IgG Alexa 594 (Invitrogen) were used. Digital images were acquired using an Axioskop2 Plus fluorescence microscope, equipped with an AxioCam CCD camera, and AxioVision 3.1 imaging software (Carl Zeiss). An 8-oxo-dG index was calculated as immunofluorescence intensity of 8-oxo-dG per MAP2-positive area of individual cells, using software in ImageJ/Fiji^44^. Confocal images were obtained using an inverted LSM700 META confocal microscope (Carl Zeiss) and were compiled using software in ImageJ/Fiji.

### Immunohistochemistry

Tissue preparation and immunohistochemical analyses were performed as described previously^16^. Coronal sections 40 μm thick were cut from 10-week-old male mouse brains using a cryostat and collected as free-floating sections in PBS. These sections were then blocked in Block Ace (Dainippon Pharmaceutical, Osaka, Japan) with 10% normal rat serum. Then sections were incubated with a primary antibody (anti-MTH1 or anti-OGG1) diluted in 10% Block Ace. Sections were processed using the Vector ABC kit (Vector Laboratories) with the appropriate biotinylated secondary antibody. Then, the 3,3′-diaminobenzidine/nickel reaction (Vector Laboratories) was used to visualise the bound secondary antibody. Digital images were acquired using an Axio Imager A1 microscope, equipped with an AxioCam CCD camera, and AxioVision 4.8 imaging software (Carl Zeiss). Views of entire coronal sections were obtained using a Nikon Eclipse 80i microscope with a Virtual slice module in Stereo Investigator software (MBF Bioscience).

### Analysis of neurite outgrowth and arborisation

The morphological stage of cortical neurons was quantified at 2 DIV using projected images obtained from confocal stacks of MAP2/DAPI stained cells. We used the following criteria for quantification, as previously described for embryonic-derived neurons^26^: stage 1, neurons lacking neurites; stage 2, with one or more minor neurite; stage 3, with one neurite at least twice as long as any other. Neuritic arborisation was quantified at 5 DIV using Sholl analysis, in which projected images of individual neurons obtained by confocal MAP-2 immunofluorescence microscopy were binalised, and traced with the centre of the soma as a focal point. Confocal image stacks were obtained using an inverted LSM700 META confocal microscope (Carl Zeiss) with 20× and 63× oil objective lenses and were collected, measured and compiled using software in ImageJ/Fiji.

### Live cell imaging

For live cell image analysis, cells were cultured in dishes with glass bottoms (Greiner Bio-one). To measure MMP, cells were incubated with 5 μg/ml JC-1 (Life Technologies) for 15 min at 37 °C. To analyse mitochondrial superoxide production, cells were incubated with 0.5 μM MitoSOX together with 0.32 μM MitoTracker Green (Life Technologies) for 20 min at 37 °C. Cells were washed with Hibernate A Low Fluorescence (BrainBits, USA) and live images were obtained using an inverted LSM700 META confocal microscope with a 63 × oil objective, and a pinhole set to a 4.2 μm optical slice. Cells were excited with a minimum laser power at an appropriate wavelength, 488 nm for JC-1 and MitoTracker Green, and 515 nm for MitoSOX. Red/green ratios for JC-1 fluorescence in a cell body and neurites were calculated in a green fluorescence-positive area using software in ImageJ/Fiji. A MitoSOX index was calculated as MitoSOX intensity per MitoTracker Green-positive area using software in ImageJ/Fiji.

### Statistical analysis

Statistical analysis was performed using JMP 11 (SAS Institute). The statistical significance between groups was determined using parametric and nonparametric methods according to data distribution, as described. *P* values < 0.05 were considered statistically significant.

## Additional Information

**How to cite this article**: Leon, J. *et al.* 8-Oxoguanine accumulation in mitochondrial DNA causes mitochondrial dysfunction and impairs neuritogenesis in cultured adult mouse cortical neurons under oxidative conditions. *Sci. Rep.* **6**, 22086; doi: 10.1038/srep22086 (2016).

## Supplementary Material

Supplementary Information
