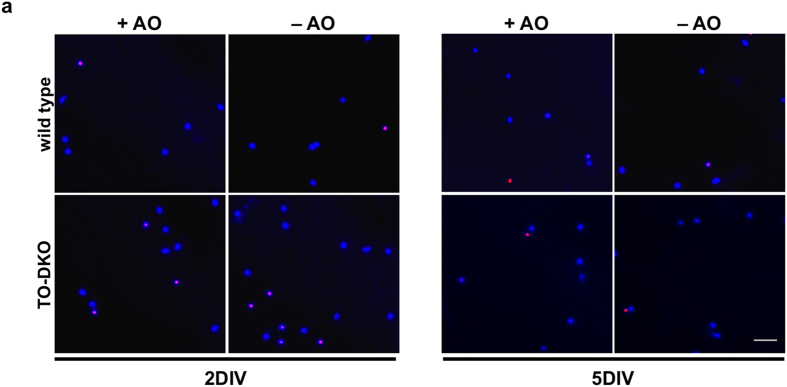# Corrigendum: 8-Oxoguanine accumulation in mitochondrial DNA causes mitochondrial dysfunction and impairs neuritogenesis in cultured adult mouse cortical neurons under oxidative conditions

**DOI:** 10.1038/srep24696

**Published:** 2016-04-29

**Authors:** Julio Leon, Kunihiko Sakumi, Erika Castillo, Zijing Sheng, Sugako Oka, Yusaku Nakabeppu

Scientific Reports 6: Article number: 2208610.1038/srep22086; Published online: 02252016; Updated: 04292016

This Article contains an error in Figure 5a. The image depicting TO-DKO cultured for 5DIV (+AO) is a duplicate of wild-type cultured for 5DIV (+AO). The correct Figure 5a appears below as Fig. 1.

## Figures and Tables

**Figure 1:**