# Supplementary material for: 8-Oxoguanine accumulation in mitochondrial DNA causes mitochondrial dysfunction and impairs neuritogenesis in cultured adult mouse cortical neurons under oxidative conditions
Source: Sci Rep. 2016 Feb 25;6:22086. doi: 10.1038/srep22086 (PMC4766534; doi:10.1038/srep22086)
Supplement: Supplementary Information [file srep22086-s1.pdf]

Supplementary Material

8-Oxoguanine accumulation in mitochondrial DNA causes mitochondrial dysfunction and impairs neuritogenesis in cultured adult mouse cortical neurons under oxidative conditions

Julio Leon, Kunihiko Sakumi, Erika Castillo, Zijing Sheng, Sugako Oka and Yusaku Nakabeppu

Division of Neurofunctional Genomics, Department of Immunobiology and Neuroscience, Medical Institute of Bioregulation, Kyushu University, 3-1-1 Maidashi, Higashi-ku, Fukuoka 812-8582, Japan

Figure Legends

Supplementary Figure S1. Localisation of MTH1 and OGG1 proteins in adult mouse brain. (a) Immunohistochemical detection of MTH1 in wild-type and TO-DKO brains. (b) Immunohistochemical detection of OGG1 in wild-type and TO-DKO brains. Scale bar = 1 mm. Both MTH1 and OGG1 are abundant in neocortex, hippocampal formation (HPF) and hypothalamus and subregions of the thalamus. In neocortex, MTH1 is homogeneously expressed in layers 2–3 and 4–6, while OGG1 is rather heterogeneously expressed in layers 2–3 and 4–6, mainly in the retrosplenial (RS), piriform (PIR) and amygdala (COA) areas.

Supplementary Figure S2. Immunoreactivity of nuclear 8-oxo-dG in TO-DKO and wild-type cortical neurons shows no differences regardless of the presence or absence of antioxidants. 8-Oxo-dG immunoreactivities (green) were detected in MAP2-positive neurons (red) by immunofluorescence microscopy. Fixed neurons pre-treated with RNase were subjected to denaturation with 2 N HCl before reacting with the anti-8-oxo-dG antibody. Adult cortical neurons isolated from TO-DKO and wild-type mice were cultured for 2 days in the absence (−AO) or presence (+AO) of antioxidants. Orthogonal views are shown to demonstrate the nuclear localization of 8-oxo-dG immunoreactivity. Scale bar = 10 μm.

Supplementary Figure S3. FCCP, an uncoupler of mitochondrial oxidative phosphorylation, inhibits neurite regeneration in adult cortical neurons isolated from wild-type mice in the presence of antioxidants. (a) Adult cortical neurons isolated from wild-type mice were cultured for 2 days in the presence of antioxidants

in B27 supplements, and were subjected to MAP2-immunofluorescence microscopy. Representative merged images are shown. Green: MAP2; blue: DAPI. Scale bar = 50 μm. Regenerating neurons were classified into three stages: stage 1, lacking neurites; stage 2, with one or more minor neurite; stage 3, with one neurite at least twice as long as any other. (b) Distribution of regenerating neurons. The percentage of neurons in each stage is shown. Error bar = SEM. More than 41 neurons in each culture condition were examined. Fisher's exact test: **, $p < 0.0001$ vs. control; *, $p < 0.005$ vs. control.

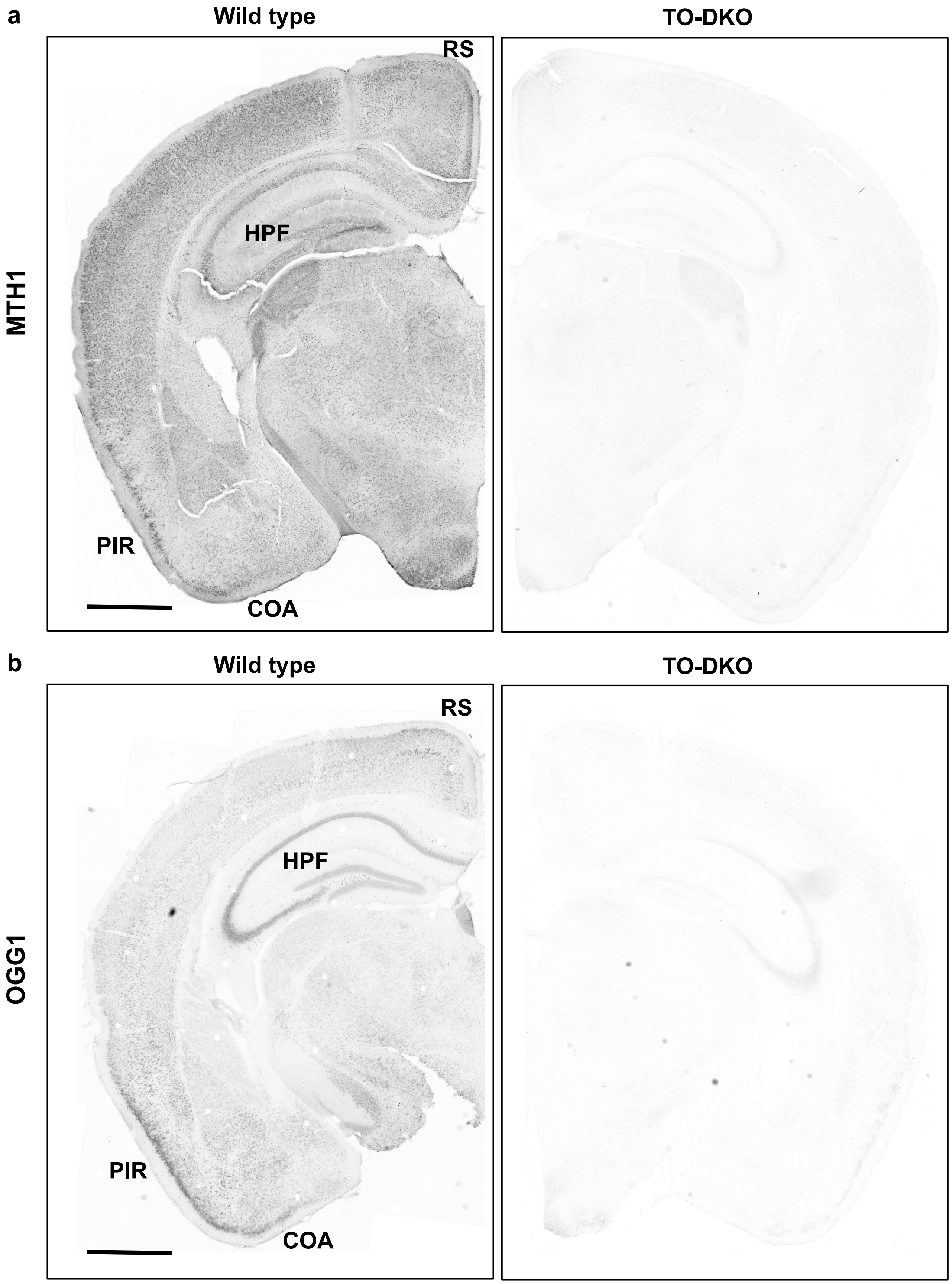

Supplementary Figure S1

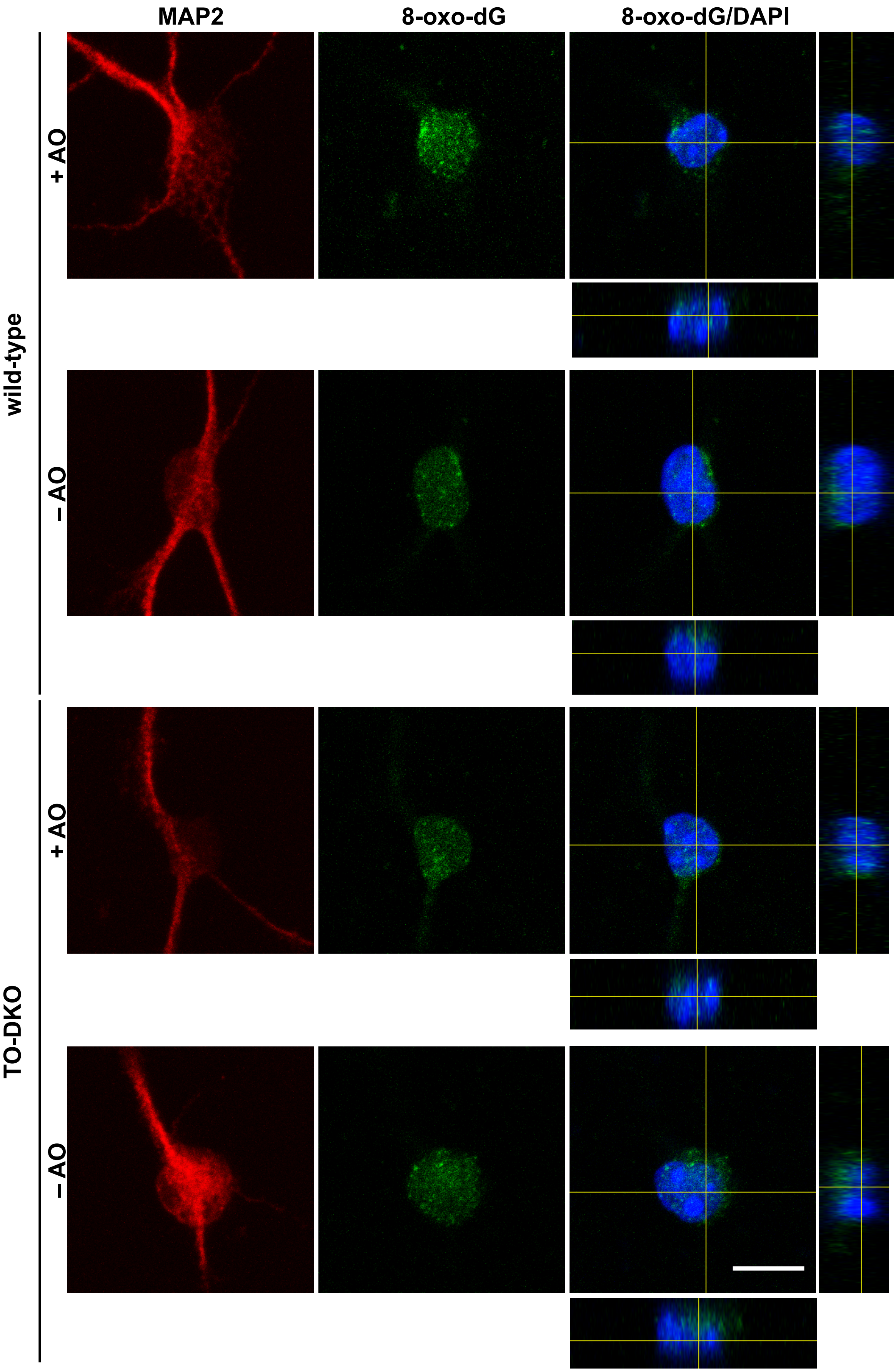

Supplementary Figure S2

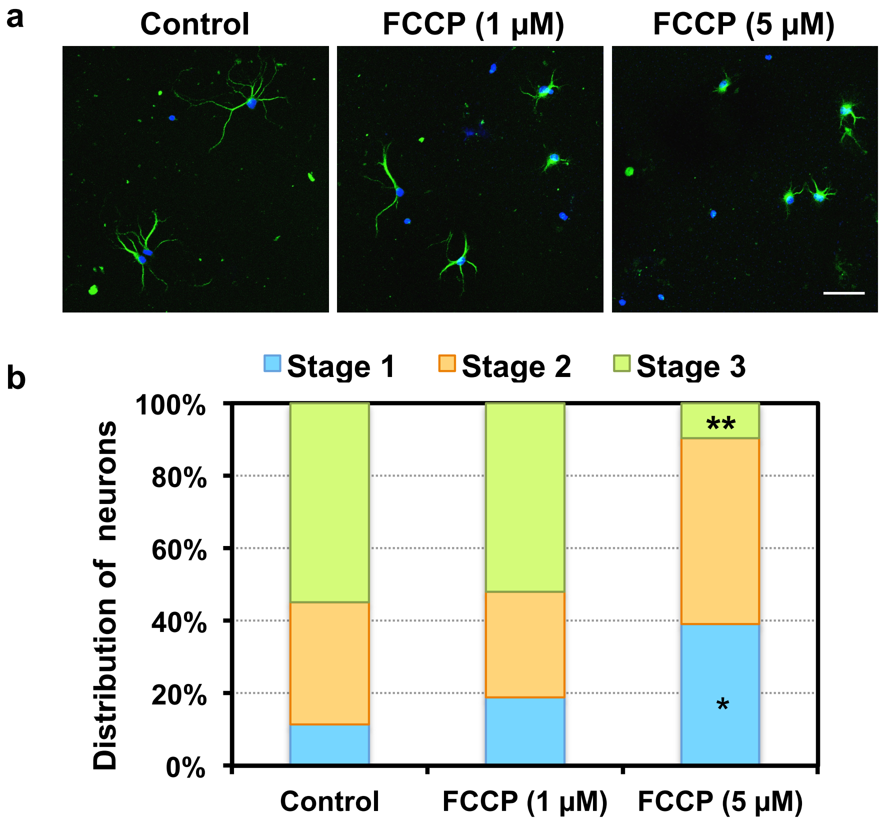

Supplementary Figure S3
